# Beyond the genome: community-level analysis of the microbial world

**DOI:** 10.1007/s10539-012-9357-8

**Published:** 2012-12-15

**Authors:** Iratxe Zarraonaindia, Daniel P. Smith, Jack A. Gilbert

**Affiliations:** 1 Argonne National Laboratory, Institute for Genomic and Systems Biology, 9700 South Cass Avenue, Argonne, IL 60439 USA; 2 IKERBASQUE, Basque Foundation for Science, 48011 Bilbao, Spain; 3 Department of Ecology and Evolution, University of Chicago, 5640 South Ellis Avenue, Chicago, IL 60637 USA

**Keywords:** Spatiotemporal sampling, Next generation sequencing, Metagenomics, ‘Omic approach, Community dynamics, Microbial community analysis

## Abstract

The development of culture-independent strategies to study microbial diversity and function has led to a revolution in microbial ecology, enabling us to address fundamental questions about the distribution of microbes and their influence on Earth’s biogeochemical cycles. This article discusses some of the progress that scientists have made with the use of so-called “omic” techniques (metagenomics, metatranscriptomics, and metaproteomics) and the limitations and major challenges these approaches are currently facing. These ‘omic methods have been used to describe the taxonomic structure of microbial communities in different environments and to discover new genes and enzymes of industrial and medical interest. However, microbial community structure varies in different spatial and temporal scales and none of the ‘omic techniques are *individually* able to elucidate the complex aspects of microbial communities and ecosystems. In this article we highlight the importance of a spatiotemporal sampling design, together with a multilevel ‘omic approach and a community analysis strategy (association networks and modeling) to examine and predict interacting microbial communities and their impact on the environment.

## Introduction: community concepts and approaches

Microbes include Bacteria, Archaea, single-celled members of the domain Eukarya (i.e. algae, some fungi, and protists), and viruses. They are all members of the biological consortia responsible for the global biogeochemical cycling that sustains all life on Earth. As such, changes in the structure of these microbial communities (species abundance and their distributions) affect the functional dynamics of whole ecosystems by influencing the ecosystem processes (biological, chemical, and physical) through metabolic feedback. Changes in the abundance of the smallest organisms can thus influence the vitality and success of the largest organisms in ways so complex that we are only now beginning to develop appropriate methods and technologies that help to characterize and predict them. The new tools are enabling us to gain a better understanding of the microbial taxonomic diversity and community function across the planet, so that we can answer the questions “Who is there?” and “What are they doing?” This basic approach, which has many parallels with the nineteenth-century natural history assessment of microbes, will eventually enable researchers to predict ecosystem changes in the micro-biosphere and determine how such changes influence global processes, such as climate.

Community-level characterization of microbes is vital because they do not exist in the defined species populations that we have come to understand from observing animals and plants. For example, if we wanted to explore the ecology of the African savannah, it would be pertinent to ask questions regarding the population demographics of lions, hyenas, zebras, and antelopes. Specifically, determining the age, sex, and health of each population is often useful for exploring and predicting the ecological impact of changes in the abundance of each taxon. Armed with this information, analyses can be carried out to determine the impact of each individual organism’s phenotype on the population’s phenotype, and to identify how specific population phenotypes will interact to affect ecosystem equilibrium and community stability. However, outside the etiology of disease, where the epidemiology and biogeography of specific pathogens are studied to understand the effects on hosts, most microbial ecology is based on community-level analysis; that is, the conglomeration of populations that define an assemblage. At this level of investigation, the tens of thousands of individual populations (and hence species) present in an ecosystem (e.g. a gram of soil) are examined to determine the community-level phenotype and its impact on ecological dynamics. Instead of analyzing factors such as a population’s age and sex ratio, predicting the ecological impact of changes in microbial abundance involves the recognition of the different interaction types among all the individual organisms (symbiotic, mutualistic, antagonistic, competitive, predatory, etc.) that govern, together with the abiotic factors of a particular environment, the presence or absence of taxa, their abundances, and their metabolism in a given habitat or community.

The concept of ‘community’ has been interpreted in a variety of ways (Odenbaugh 2007). In plant and animal ecology, communities are defined as multispecies assemblages, in which organisms live together in a well-defined environment and interact with one another (Konopka 2009). However, defined boundaries are not particularly easy to apply to, for example, an ocean or terrestrial ecosystem. Therefore, some researchers refer to the community as a supra-organism with “emergent properties” (explained below), where the boundaries of a community are defined by the strength of organismal interactions rather than physical boundaries (Levins and Lewontin 1985).

In microbial ecology difficulties in defining “boundaries” and “interactions” hamper a unique interpretation of community even when researchers study the microbial structure of the same ecosystem. The delimitation of microbial communities based on environmental boundaries is complicated because there is an extremely rapid feedback among microorganisms and environment, with both being constantly influenced and changed in a cause-effect cycle. This constant to-and-fro between environment and microbial community can lead to rapid community turnover, and creates microhabitats at small spatial and temporal scales. In the microbial world it is not clear, therefore, whether the individual microenvironments should be considered as the boundaries of microcommunities inside higher biological assemblages called communities, or whether they should be considered as communities on their own. It is arguably a philosophical issue, but one of vital importance when we consider how to model these systems. Defining an empirical community can be even more complex in highly fluid dynamic systems, such as oceans, where dynamic tides and currents mean microorganisms can be transported rapidly from one habitat to another. Microorganisms might then be temporarily part of a water sample because of chance events. They might persist in that environment for only a brief time, or they might interact with the individuals that were already present in that niche, thereby altering that community. It is not obvious, therefore, that all the individuals present in a sample from a single time point should be considered part of a community, especially when their presence is potentially the result of stochastic and temporary events. In addition, answering the question of what defines an interaction is complex in microbial ecology. For example, a one-liter sample of water from the surface (0–2 m) of the Atlantic Ocean needs to be considered in relation not only to the organisms present in that sample, but also those that are not present in that sample but which interact metabolically (due to the distribution potential of metabolites released by a cell) with the sampled community. At best, therefore, a sampling event can be considered an incomplete snapshot of a potential community.

Recently, Konopka (2009) defined a community as a system exhibiting characteristics that each of its component organisms don’t have when analyzed in isolation. This addresses the so-called emergent properties of the community, in that the community has a property that occurs because of, and is different from, the properties of its individual components. Some of the issues to do with the emergence of these properties are reviewed by Corning (2002), who proposed that multidimensional interactions between the biological, physical, and chemical components of a system have cooperative effects that play a major causal role in the evolution of biological complexity. This multi-dimensional complexity is the cornerstone of the ecological niche concept, and as such, provides a rational foundation for describing a community as a system comprised of many niches, but one that also presents its own specific niche. In this sense, Doolittle and Zhaxybayeva (2010) argued that communities constitute lineages that occupy particular niches and that they can migrate collectively (sometimes as a biofilm) to new environments where they reestablish their niches. Thus, the composition of a microbial community might be selected for its collective adaptation (where adaptation is an emergent property of the community) to environmental factors.

Several studies that used artificial selection experiments have suggested that the phenotypic traits of a community are heritable and that selection can operate above the level of the individual organism and even population. Swenson et al. (2000a) selected aquatic ecosystems for the ability to degrade the environmental pollutant 3-chloroaniline in four replicated lines, and compared them with four lines in which parent ecosystems were randomly chosen without respect to their degradation ability. Over many generations of variation and selection, three of the four selected lines increased their degradation ability whereas none of the four non-selected lines did so (Swenson et al. 2000b). In addition, several host-associated communities have shown heritability, such as when the reciprocal transplant of the gut communities of mice reproduces the function of the transplanted community in the new host (Vijay-Kumar et al. 2010).

This point of view can also be applied to the concept of reproduction, in regard to whether an individual microbe is the reproductive unit or whether communities should be understood as a higher level of reproductive entity (Godfrey-Smith 2009). For example, each human is an assemblage of human cells (10 trillion) and many more microbial cells (100 trillion). Each person could be conceptualized as a human-microbial ecosystem, with the whole system (meta-organism) responding as a single reproductive unit. Some research suggests that microbes could be responsible for mate selection in fruit flies (Sharon et al. 2010), which suggests that the microbial part of a meta-organism may play a significant role in the behavior and evolution of the host organism. However, we tend to ignore this potential influence when we consider the reproduction of an organism, choosing to see the host as a whole unit rather than a collection of host and microbial cells, and avoiding the fact that each component of the meta-organism has its own level of influence over the event. This simplification (i.e. by just taking the emergent property of the system) might be a useful model when considering microbial communities in an environmental sample. In this respect, the community may be conceptualized as the unit of ‘reproduction’ and the point of action for natural selection.

## Amplicon metagenomics and shotgun metagenomics

That microbes do not exist as isolated taxa might seem like a scientific dogma now, but historically microbes have been analyzed by isolating them in axenic culture (a pure culture of microorganisms free from cells or living organisms of any other species) on artificial media. This approach led to a narrow picture of the diversity of an ecosystem as only an estimated 5 % or less of the microbial diversity in the biosphere is thought to be cultivable with standard culturing techniques (Amann et al. 1995; Curtis 2002). Moreover, since Bacteria, Archaea, and microbial eukaryotes do not live in single species ecosystems, axenic culturing limits our ability to examine the interactions between microbial species, and to understand the species-habitat and community-habitat interactions.

A new era of microbial ecology was initiated with the concept of cloning DNA directly from the environment, which is commonly attributed to Pace et al. (1986), and was applied by Schmidt et al. (1991) who characterized 16S rRNA sequences from a Pacific Ocean picoplankton population by cloning environmental DNA into a phage genome and screening for clones that contained 16S rRNA genes. By 1998, this technique of randomly cloning environmental DNA followed by elaborate screening methods became known as metagenomics (Handelsman et al. 1998), which can be translated as ‘beyond the genome’ (Gilbert and Dupont 2011). This new label referred to the concept that researchers were now exploring the genomic DNA from all the genomes of all the organisms in an environmental community through cultivation-independent methods, and, therefore, going beyond the single genome. More recently, with the advent of high-throughput sequencing strategies, metagenomics has diverged into two fields: amplicon metagenomics (sequencing of libraries of a PCR-amplified gene of interest), and shotgun metagenomics (screening or sequencing of libraries of randomly isolated DNA fragments). Here we discuss these concepts and provide some historical perspective.

Typical modern-day metagenomic projects assess the microbial diversity of different environments by assembling a catalog of the discovered species and their functions. These studies seek primarily to characterize the taxonomic structure of the microbial community, either by using a taxonomic marker gene such as the 16S rRNA (i.e. amplicon metagenomics) or by using random shotgun sequencing methods. The shotgun metagenomic approach can be further divided into two technically distinct groups. (1) cloning-based approaches using vectors such as fosmids (single-copy circular DNA vector of bacterial origin capable of replicating autonomously), cosmids (multi-copy DNA vector of bacterial origin with autoreplication capability), and bacterial artificial chromosomes (BACs) suitable for cloning large-insert (10–100 Kbp) libraries, or the use of plasmid vectors for short-insert (<5 Kbp) libraries. These vector-based DNA isolation techniques are designed mostly for heterologous expression and sequencing, as well as direct isolation and sequencing approaches. (2) Direct next-generation sequencing (NGS) approaches that forego traditional cloning strategies for DNA isolation, relying instead on DNA fragmentation and capture via various ligation strategies (Gilbert and Dupont 2011).

Continuing advances in NGS technologies have resulted in dramatically faster and cheaper sequencing of DNA libraries. The current pinnacle of this acceleration is the ability of shotgun metagenomics to reconstruct whole bacterial and archaeal genomes without prior knowledge of these organisms (or their genome sequence) by using powerful assembly algorithms that join short overlapping DNA fragments generated by the sequencer into longer contiguous sequences (Thomas et al. 2012). The two most commonly applied sequencing platforms in current metagenomic studies are the 454/Roche pyrosequencing technology and the Illumina/Solexa system. 454/Roche pyrosequencing is based on emulsion-PCR to amplify random DNA fragments clonally, which are then attached to microscopic beads and sequenced. This technology can currently be used to generate approximately a million 500–800 bp length reads (sequences) per run for ~$10,000. The Illumina/Solexa technology is based on the sequencing-by-synthesis technology of molecules bound to a surface (cell flow) where clonal clusters are generated through bridge amplifications (see Metzker 2010 for a more detailed explanation of both methods). Currently, Illumina platforms (e.g. HiSeq2000) can generate 2–3 billion 150 bp length reads for ~$20,000.

This disparity in sequencing depth (number of reads per run) and read length generated by each platform has led researchers to use primarily Illumina for amplicon metagenomics and 454-pyrosequencing for shotgun metagenomics. The short-read length, but high depth, of the Illumina platform is well suited to amplicon metagenomics, such as in studies where the hyper-variable regions of the 16S rRNA are analyzed. In those studies, extraordinary coverage of community composition can be obtained, resulting in the recovery of huge number of species from an environmental sample (e.g. Caporaso et al. 2011a, 2012b). In contrast, the 454 pyrosequencing platform, which generates fewer but longer reads, is advantageous in shotgun metagenomics as these longer reads enable better functional assignments of sequenced gene fragments. Genome assembly projects have found that a combination of both 454 and Illumina is optimal for attaining both deep coverage (number of times a particular genomic region is sequenced) and robust read-through of repetitive regions of genomes. However, with the Illumina MiSeq platform now generating ~10 million 450 bp sequences per day for ~$2,000, this desktop-sized sequencer may prove itself capable of providing the balance between read depth and read length and thus be suitable for most metagenomic studies.

Next-generation sequencing platforms also offer the flexibility to examine either one sample in extreme depth, or to explore hundreds to thousands of samples in parallel via multiplexing. This is done by adding a 9–12 bp DNA tag to each DNA fragment prior to sequencing, then using that tag to identify the sample that each fragment came from when many samples have been physically pooled together (multiplexed) and sequenced in the same run. This method permits the simultaneous exploration of hundreds or thousands of bacterial communities in a highly cost-effective manner (Knight et al. 2012).

## Microbial community diversity and phylogeography

The terms “community” and “assemblage” in the context of metagenomic studies refer to the collection of microbial genomes in an environmental sample; that is, all the microbes present in a sample are considered the microbial community of that particular environment, irrespective of whether all the potential interacting units that define the extent of the community are actually represented. To date, amplicon and shotgun metagenomics have been used to characterize the taxonomic structure and functional potential for microbial communities in hundreds of environments, including marine sites (Gilbert et al. 2008, 2009; Rusch et al. 2007; Venter et al. 2004), agricultural soils (Rondon et al. 2000), forest soils (Lee et al. 2004), and extreme environments such as acid mine drainage channels (Tyson et al. 2004), hypersaline waters (Narasingarao et al. 2011), and permafrost (Mackelprang et al. 2011; Yergeau et al. 2010).

Striking results have been reported from studies investigating the relative abundance of taxa in different environments. For example, even with the extraordinary depth of next-generation sequencing technologies, virtually none of the bacterial communities studied have been completely sequenced; this speaks to the incredibly high diversity of environmental samples. For example, in a gram of soil, there are approximately a billion microbial cells, containing an estimated 4 petabase pairs of DNA (4 × 10^12^ bp). But even with today’s sequencing technology it is financially unfeasible to sequence all four petabases once (approximate cost = $150,000); indeed, most studies will generate only a few billion base pairs per sample. This number corresponds only to a tiny fraction of the actual genomic content of the community, and results in data sets representing primarily the more abundant organisms. Adding to the level of community diversity in an environment is the fact that microbial community structure can change dramatically over both spatial and temporal scales as influenced by changing environmental conditions. For example, studies of the human mouth have shown that different surfaces of the same tooth have distinguishable microbial communities (Zaura et al. 2009), while microbial communities in the open ocean may be stable across many kilometers. Because significant temporal changes have been found in a variety of microbial communities (Brown et al. 2009; Caporaso et al. 2011b, 2012a, b; Fuhrman 2009; Gilbert et al. 2012), spatial analysis on its own is not sufficient to understand microbial community dynamics. Therefore, studies should make use of both longitudinal and cross-sectional sampling (spatiotemporal sampling) in order to collect a complete representation of the real assemblage composition (extent of organism interaction) for a community.

Microbial communities have been characterized by a few dominant taxa, and many low-abundance, highly-diverse taxa. This latter group has been referred to as the “rare biosphere” (Pedrós-Alió 2007; Sogin et al. 2006). Interestingly, while a biosphere is normally used to describe a self-contained system, acquiring connotations similar to the community term, there is little evidence to suggest that these collections of rare bacteria are self-contained. Indeed, it is more likely that they are continually interacting with more abundant organisms and changing in abundance themselves. As community member abundance can change on different temporal scales, a taxon once considered rare could become, or may have been, an abundant taxon at another time (Brown et al. 2009; Caporaso et al. 2012b; Gilbert et al. 2012).

Much remains to be learned about this rare biosphere, as for example, it is not clear whether some groups are rare in every environment; that is, if “rareness” is an evolutionarily conserved mechanism to avoid predation (Reid and Buckley 2011). Rare taxa in an ecosystem may carry out key physiological functions, be responsible for the resilience of a community, and serve as a reservoir of genetic resources that can provide novel material to the community (Fuhrman 2009; Patterson 2009; Reid and Buckley 2011). For example, *Leptospirillum ferrodiazotrophum,* which accounts for less than the 10 % of the community biomass in a highly acidic mine drainage, is solely responsible for nitrogen fixation in that habitat (Tyson et al. 2005). In the marine realm, recent evidence suggests that taxa representing 0.01 of the total marine bacteria fix more nitrogen than do the larger organisms (Montoya et al. 2004). Rehman et al. (2010) found that phylotypes (defined as organisms with 97 % genetic similarity) showing the highest activity in the gut mucosa microbiota represented less than 1 % of overall community. These examples highlight the fact that low-abundance taxa can potentially be more active in the environment than abundant species.

The rare biosphere may also be composed of dying or dead cells that function as reservoirs of genetic material, and thus might play a role in adaptation if living cells are able to acquire this genetic material (Reid and Buckley 2011). To ensure that the rare biosphere is not an artifact derived from sequencing error or inappropriate sampling design, and to understand the role of these microbes in the environment, we need to combine several strategies. Metagenomics alone, at a modest sequencing depth, cannot provide a highly resolved view of the community structure because of its bias toward sequences of the most abundant taxa. To establish what proportion of the rare biosphere might be ‘alive’, research combining sequencing effort with methods such as fluorescence in situ hybridization (FISH) to visualize rare cells, cell sorting to capture them, and metatranscriptomics (sequencing of the expressed genetic material) and metaproteomics (exploration of the proteins expressed and folded by the community) to examine their functional activity, may offer a better understanding of the role that the rare biosphere has within the larger microbial community.

Metagenomic studies have suggested that environments contain not only species adapted to that specific ecosystem, but also species that have arrived in the area and survived but do not flourish. These organisms represent the latent seed bank (Caporaso et al. 2012b; Fuhrman 2009; Fuhrman et al. 2008; Gilbert et al. 2009; Lennon and Jones 2011; Pedrós-Alió 2006; Sogin et al. 2006). The seed bank hypothesis suggests that microbes can enter a dormancy state of low metabolic activity and flourish again when the environmental conditions change. For example, Caporaso et al. (2012b) analyzed samples from the English Channel, comparing a single water sample analyzed at a depth of 10,000,000 16S rRNA sequences against samples collected every month for 6 years and analyzed at a depth of 10,000 16S rRNA sequences. They found that 99.95 % of all taxa present in the six-year survey were also present at the single deeply sequenced time-point, and that the sum total of species richness found in the 72 monthly observations comprised less than five percent of the total diversity in the deeply sequenced time point. These results suggest that the differences in community composition observed between time points in the 72-month observation were due to changes in the relative abundance of taxa that were always present in the environment, rather than fluctuations in community membership, and that the coastal pelagic ecosystem, despite being a fluid dynamic environment, maintains a persistent microbial community.

However, if longitudinal sampling in a coastal marine ecosystem suggests that all the organisms are always present, then what role do differences in the distribution of taxa abundances between environments play in our ability to interpret these ecological patterns? While the abundances of most bacterial phyla vary among habitats (soil, freshwater, oceans), a few phyla appear to be present in many environments (e.g. figure 9.7 in Kirchman 2012). For example, Proteobacteria are observed to be common to most environments even though different ‘classes’ of this group dominate soil, fresh water, and oceans. There are also examples of environmentally restricted phyla, like the Acidobacteria, which are common in soils but rare in freshwater and marine environments. Similarly, Actinobacteria are abundant in soils and fresh water, but are rarely present in marine samples. Tamames et al. (2010) used 16S rRNA sequences from several experiments in natural and artificial sources to study the environmental distribution and diversity of prokaryotic taxa. Their results showed that most taxa can be found in many environment types and that while environmental specificity is not very common at the higher taxonomic levels (phylum to family), it emerges at lower taxonomic levels (genus and species). The most selective environments were those of animal tissues and regions of extreme temperature, while soil and freshwater habitats were found to be less restrictive environments.

While it is expected that habitats that differ in physicochemical features will vary in their microbial community structure, it is more controversial to respond to the question of whether two microbial communities will differ in two geographically separated locations that have the same environmental conditions. In 1934, Lourens Baas-Becking captured this idea in his famous quote, “everything is everywhere, but the environment selects” (reviewed in de Wit and Bouvier 2006). He proposed that the small size and extremely rapid dispersal potential of bacteria meant that they had the potential to exist everywhere across the globe. Their biogeography, therefore, would be dictated by the environmental conditions they encountered when they arrived to a new environment. According to this view, historical factors such as isolation and geography will not be the forces determining microbial distribution. There are numerous studies that have generated data either in favor of or against this idea. For example, bacterial communities in similar soil environments are similar even in different latitudes (Fierer and Jackson 2006). In a similar way, the same 16S rRNA and carbon and sulfur metabolism genes from *Zoothmnium niveum* are found in the Mediterranean and the Caribbean seas (Rinke et al. 2009). These examples suggest that local environmental parameters are selecting for these bacteria as they arrive in these locations. Similarly, evidence of the persistent microbial seed-bank (Caporaso et al. 2012b) also suggests that a large number of the bacteria in a given ecosystem are already present, albeit at extremely low abundance, and potentially in a latent form.

In contrast, Martiny et al. (2006) examined a number of microbial biogeography studies to conclude that they indicated that the environment is only partly responsible for the spatial variations found in microbial diversities and that everything is not, therefore, everywhere. This is corroborated by studies that have found that geographical isolation affects the genetic diversity of microorganisms, and that there is a non-random distribution of microorganisms even in samples separated by only few kilometers (Papke et al. 2003; Whitaker 2003). In these cases, distance effects are negligible in contrast to environmental effects. Besides, Green and Bohannan (2006) demonstrated evidence of taxa-area relationships where community differences did increase with distance. Galand et al. (2009) studied the biogeography of the rare biosphere in the Arctic Ocean and found that rare taxa experienced the same ecological drivers, and present the same patterns, as abundant taxa. This suggests that ‘rare’ bacteria, at least at the depth of sequencing observation used in that study, may not be part of a latent seed-bank, but are actually active members of the community.

While evidence for both sides of the “is everything everywhere?” debate mount up, it is becoming increasingly clear that current methods may be incapable of definitively answering this question. The reason for this is two-fold. First, it is virtually impossible to show conclusively that a microbial taxon is absent from a given location with current sequencing methods, and because metagenomics is biased towards more abundant species, rare taxa are usually not included in these analyses (Green and Bohannan 2006). Second, most diversity studies rely on 16S rRNA sequences at a 97 % similarity level, which represents phylogenetic diversity but does not measure the full genomic diversity that can vary considerably between closely related taxa.

## Limitations of metagenomics approach and its implications for defining “species”

Despite the remarkable scope of metagenomics, it still has many shortcomings. In metagenomics, the concept of “species” has been substituted by Operational Taxonomic Units (OTUs), which describes organisms with higher than 97 % 16S rRNA sequence similarity as belonging to the same species. However, the definition of species based on this arbitrary nucleotide identity cutoff is controversial, because although it is generally accepted to be true that two organisms that share less than 97 % nucleotide identity between their ribosomal RNA small subunit genes do not belong to the same species, the opposite is not always a valid statement. For example, three species belonging to the genus *Bacillus* that share >99 % 16S rRNA nucleotide identity are considered as separate species because of their physiological, and hence genotypic/phenotypic, differences (Vilas-Bôas et al. 2007). Moreover, there is some evidence for horizontal transfer of 16S rRNA genes between different species (Schouls et al. 2003), which would lead to misleading inferences in phylogenetic trees constructed on the basis of these genes (Gevers et al. 2005).

In spite of these problems, amplicon metagenomics has been widely used to characterize bacterial “species” abundance and distribution patterns across thousands of ecosystems using the 16S rRNA genes as a taxonomic marker; e.g. the Earth Microbiome Project (Gilbert et al. 2010a, b; Gilbert and Dupont 2011). One potential solution to this problem is to create studies that use more than one gene. Although this is a more expensive approach, the financial disadvantage may be outweighed by the ability to capture a more accurate picture of the whole genome diversity of a community. Genomic diversity, even in terms of gene complement, can be extraordinary even within the same species. For example, three strains of *E. coli* have been shown to only share 40 % of their genes (Welch et al. 2002). The concept that there are genes common to the genomes of all strains of a species, and genes that are either partially shared or unique for each strain, has led to the terms ‘core genome’ and ‘accessory genome’ respectively (Medini et al. 2005; Tettelin 2005; Mira et al. 2010). The accessory genome may have been differentially retained in the strains from the common ancestor as an adaptation mechanism to changing environmental conditions or may have its origin in lateral gene transfer, also called horizontal gene transfer (Ochman et al. 2000). The concept of core- and accessory-genomes has been combined into the ‘pan-genome’ theory, which describes the full complement of genes in a species. The idea of a pan-genome for a species suggests that the genomes of multiple independent strains will be required to understand the diversity and complexity of any bacterial “species”. This is a level of genomic resolution that metagenomics, at current technically achievable sequence depths, may not be able to provide. However, it is believed that the use of ‘single cell’ genomics (Woyke et al. 2010) could yield extensive information about the genomic variability of a bacterial population and could lead to improved differentiation of bacterial lineages on the basis of their nucleotide polymorphisms and whole genome gene complement.

Unlike amplicon metagenomics, shotgun metagenomics seeks to elucidate the functional potential of a microbial community. While shotgun metagenomics has considerable advantages over amplicon metagenomics (e.g. it does not involve PCR amplification or primer biases), it also has notable limitations. Firstly, studies have reported that the abundance of taxa and their functional genes in a metagenomic library vary depending on the DNA extraction protocol used to acquire the nucleic acid from the environmental sample (Morgan et al. 2010; Delmont et al. 2012). This is a problem for all nucleic acid studies, but must be taken into consideration in metagenomic analyses if we intend to compare the community structure of the ecosystems studied. Secondly, metagenomic datasets are often only sequenced to a low depth compared with the quantity of DNA in a sample, which results in only the most dominant populations being observed. Thirdly, it is difficult to annotate the function or taxonomy of a short sequence fragment resulting in a large portion of data lacking an appropriate annotation; and finally, the lack of functional verification for sequence annotation is a persistent problem, since metagenomics sequence fragments can only be annotated if they have sequence homology to genes (already available in public databases) that correspond to biochemically characterized proteins (Warnecke and Hugenholtz 2007).

Methods to overcome these limitations rely on the combination of metagenomics with other approaches. For example, to overcome the first two limitations, Warnecke and Hugenholtz (2007) suggest dividing microbial communities into simpler subsets by cultivating organisms of interest (although it is often difficult or even impossible to grow the environmentally relevant organisms), or to carry out enriched population studies where researchers try to preferentially target the population of interest removing the rest of the taxa (e.g. by filtration, centrifugation) and accompany this strategy with single-cell methodologies such as fluorescent activation cell sorting (Brehm-Stecher and Johnson 2004) or microfluidics (Weibel et al. 2007). To improve sequence annotation, sequence fragment assembly can be used to make longer reads out of shorter reads based on overlapping regions, with the caveat that these may result in pan-gene fragments; that is, with the joining of gene fragments from multiple strains of a single species, or multiple species of a single genus combined into one fragment (Thomas et al. 2012). To improve the number of proteins with known function or taxonomy is a monumental task, and has many potential barriers to success. However, the efforts to increase the speed and efficiency by which new proteins are assigned to a relevant function will help to alleviate this gap in knowledge. A final solution will be to begin comparing metagenomic data to sequence data from metatranscriptomic and metaproteomic studies (Jansson et al. 2012). The identification of commonalities between these ‘omic data may aid researchers in clustering specific predicted proteins into new protein families that can be targeted for functional assays.

## Functional analysis in metagenomics, metatranscriptomics and metaproteomics

The principal objective of most metagenomic studies, once the taxonomic structure of a community has been characterized, has been to link a function to its phylogenetic source in order to understand what different organisms do within their communities. Numerous studies, such as the Global Ocean Survey (Rusch et al. 2007), have been carried out in an attempt to elucidate which species are involved in processes such as phosphorus, sulfur, and nitrogen cycling (reviewed in Gilbert and Dupont 2011). In addition, the discovery of organisms containing molecules and genes of commercial interest is growing rapidly. Functional analyses of metagenomic data have produced information about new antibiotics, hydrolytic and degradative enzymes, biosynthetic functions, and antibiotic resistance enzymes (Riesenfeld et al. 2004; Lämmle et al. 2007; Garmendia et al. 2012).

Unfortunately, the discovery of new functions or genes associated with as of yet uncultured taxa has been hampered by the scarcity of reference genomes available (consensus representation of the set of genes of a given species that serve as reference to assemble and annotate ‘omic data). Reference genomes are essential for accurately determining the taxonomy of short metagenomic sequence fragments (Woyke et al. 2009). While metagenomics is usually considered a gene-centric approach (i.e. focusing on the gene as a unit of investigation), emerging genome-centric methodologies such as the single-cell DNA sequencing (Marcy et al. 2007; Raghunathan et al. 2005; Stepanauskas and Sieracki 2007; Woyke et al. 2009; Zhang et al. 2006) will undoubtedly assist the assembly and annotation of gene-centric metagenomic studies.

While metagenomics identifies the potential function of a community in the environment, *metatranscriptomic* analysis (the study of the RNA from the entire community of organisms) determines which microbes are active and which genes are transcribed. Because metatranscriptomics targets only the transcribed elements of each genome, the resulting data set is less complex, and requires less sequencing depth in order to achieve the same level of coverage compared to a metagenome. However, despite this seeming advantage, metatranscriptomics faces other major technical issues that have impeded its broad application. Firstly, RNA molecules generally have a short half-life, often degrading in minutes or even milliseconds, which makes sampling the material from an environment difficult, as the act of sampling may change the metatranscriptomic profile. Secondly, ribosomal RNA (rRNA) represents the majority of the RNA extracted (Urich et al. 2008), which limits access (i.e. for a given sequence depth the majority of sequence data generated will be rRNA) to the informative messenger RNA (mRNA) molecule that reveals the genes and pathways expressed under a given condition. In response to these challenges, protocols have been developed to isolate mRNA transcripts from total RNA, including the development of subtractive hybridization (Giannoukos et al. 2012) and enzyme-based rRNA degradation (Sharma et al. 2010).

A number of studies have employed metatranscriptomics to explore environmental gene transcription. Poretsky et al. (2005) were the first to combine the enrichment of mRNA by rRNA subtracting methods with a non-targeted gene sequencing approach, in which they used random primers to amplify the mRNA and a vector-cloning step before Sanger-sequencing of the captured transcripts. This method was rapidly superseded by approaches using NGS techniques, for example pyrosequencing was initially used to sequence all the RNA (both rRNA and mRNA) from soil communities (Leininger et al. 2006; Urich et al. 2008), while subsequent approaches combined rRNA subtraction with NGS in marine environments (Frias-Lopez et al. 2008; Gilbert et al. 2008, 2010c). One of the more recent advances in the field introduced the stable isotope probing (SIP) approach (Dumont et al. 2011) to label organisms using specific substrates, so that the label was actively incorporated into their mRNA. These studies have given insights into which genes are most actively transcribed in different environments, helping microbial ecologists understand the temporal and spatial dynamics of community-level gene expression. For instance, these studies revealed that transcripts involved in RNA and protein synthesis, protein folding and export, and DNA repair are highly abundant in soil and aquatic ecosystems (Kirchman 2012). Additionally, some of the highly abundant transcripts in extremely disparate marine ecosystems were found to be identical, despite different technical approaches, suggesting ubiquity in certain functional gene transcription between very different environments (Gilbert et al. 2008).

Another way to link genomic diversity to functional activity is through *metaproteomics*, the study of all the proteins in an environmental sample. While metagenomics tells us which genes a community has, and hence which proteins it has the potential to produce, and metatranscriptomics refers to which genes are actually transcribed but not which proteins are actually translated and present, metaproteomics tells us which proteins are actually expressed and present in the community. Since it was first applied to study an acid mine drainage community (Ram et al. 2005), metaproteomics has been used to analyze more complex environments such as the human gut (VerBerkmoes et al. 2009), soil (Chourey et al. 2010), and the ocean surface (Morris et al. 2010; Sowell et al. 2011). Due to the complexity of protein extraction, separation and identification, this field has been less widely used than metagenomics and metatranscriptomics and it is still in its infancy. Challenges in the field include the reliable identification of low abundance proteins. Resolving this problem is dependent on the development of higher sensitivity mass spectrometers. Also, while there is some evidence of a relationship between the relative abundance of an mRNA fragment and the relative abundance of its corresponding protein, this work still requires significant interpretation because of the posttranscriptional regulation of transcripts.

To date, the vast majority of ‘omics studies have been organism, gene, or pathway-centric, rather than focused on the integrated community. Despite the great potential of the current methods and techniques to analyze the ecosystem at a community level, the interaction between members of a community and their link with the environmental gradients they are exposed to has been understudied (Jansson et al. 2012). One of the significant barriers is how to use different techniques to explore relationships observed by a specific ‘omic technique, or relationships between patterns observed with different ‘omics techniques. For example, comparing shotgun metagenomics and metatranscriptomics, researchers found that there was very little overlap between the mRNA transcripts and genes that could be annotated (Gilbert et al. 2008, 2010c). Similarly, it is difficult to find direct correlations from transcripts to proteins (Smith et al. 2010). However, as more datasets become available it will become increasingly easier to explore interactions between different ‘omic levels, aided by the development of novel data analysis tools to enable integration of, for example, metagenomic data and metaproteomic data, helping us to explore interactions within a community from multiple perspectives.

## Analyzing the community as a system

Recognition of the fact that organisms modify their environments and that such phenomena are important on a global scale is vital in designing the next generation of experimental and observational studies. The properties of the community, or community phenotype, as we have already suggested above, can be understood as the emergent properties of multifaceted interactions between different populations (e.g. metabolic, predatory, competitive, cooperative). These interactions are in turn driven by the interactions between each cellular unit comprising that population. Those cells are in turn driven by the metabolic interactions and molecular dynamics of their cellular biochemistry, and so on. One way to capture these multilevel interactions is via computational simulations of community dynamics. For example, genome-derived metabolic flux-balance models (Henry et al. 2010) can be used to generate a simulated community, in which the energy flux between cells can be balanced within an artificial system. In this simulated system, we can populate cellular constructs with cellular metabolic models from different species (derived from their genomes), then set this system running under defined external stimuli and observe the effects of changing environmental conditions (e.g. O_2_ or CO_2_ concentrations) on the population structure of the microbial community.

In principle, it is conceivable that the quantified inputs and outputs of a system model designed to emulate every ecosystem on Earth could drive a system model of the whole Earth, in order to enable highly accurate predictions of global responses to, for example, climate change. Doing so, however, would require, firstly, a genome for every relevant organism in a community at every given potential environmental condition for that community; secondly, a better understanding and predictive capability for genomic evolution in microbial organisms; and finally, a much more comprehensive understanding of cellular biochemistry, the functional properties of specific genes, and the transcriptional and proteomic response of each organism to a myriad of external stimuli scenarios. This information currently does not exist for any system on Earth. While the development of this knowledge and the necessary technologies should certainly be pursued, until then it is necessary to focus on how particular communities, and their collective phenotype, respond to myriad external stimuli.

Determining species interaction in a community is of great interest because it could help microbiologists understand why some organisms tend to co-occur while other species are never found together in an environment. Species interaction analysis could also help us understand and detect cooperative effects, where two or more populations or species cooperate to supply each other’s nutritional needs, often by metabolizing compounds that each species or population is unable to biodegrade alone. Co-occurrence, however, does not necessarily infer a metabolic or physical interaction. Yet co-occurrence networks (e.g. Steele et al. 2011) can generate hypotheses about potential interactions that can then be tested experimentally in regard to particular physical and chemical variables.

Regarding community-environment interaction, the integration of environmental aspects to explore community changes in previous studies has been done in an indirect and discrete way, comparing qualitatively dissimilar environments, such as terrestrial versus marine niches (Gianoulis et al. 2009). For example, Dinsdale et al. (2008) used a comparative metagenomic approach to analyze the frequency distribution of microbial and viral metagenomic sequences in order to explore the functional potential of nine environments. Currently, several endeavors are under way to help generate information about global microbial ecosystems; for example, specific natural habitats are regularly sampled and continuously monitored in order to recover time-series information.^1^ The Earth Microbiome Project (http://www.earthmicrobiome.org) is an additional multidisciplinary effort that aims to analyze microbial communities across the globe in a comparable framework (Gilbert and Dupont 2011; Gilbert et al. 2010a, b; Knight et al. 2012). These efforts further our understanding of community composition changes over time and the environmental factors that may have the greatest influence on the observed changes in microbial diversity. This information together with the future development of powerful predictive models will make it possible to forecast changes in microbial communities on the basis of existing patterns (Larsen et al. 2012).

Species interactions and how these vary with the environment can be represented by association networks or by network-modeling approaches. One of the first studies using microbial networks developed a mathematical method called a “local similarity metric” for evaluating time-lagged relationships; that is, to study if one organism tends to follow another or tends to decline after another one increases (Ruan et al. 2006). These pairwise interactions were used to construct a network diagram, which reflected the positive and negative relationships among microorganisms and between microbes and environmental variables. Fuhrman and Steele (2008) extended the analysis by focusing the network analysis on what they called “nearest neighbors” (organisms or factors that correlate directly either positively or negatively with one another). Results suggested that the taxonomic relatedness did not necessarily mean that these taxa would be found in similar ecological niches. However, a six-year time-series study in which microbial association network analysis was carried out (Gilbert et al. 2012) showed that correlation in abundances was stronger within bacterial taxa than between bacteria and eukaryotes, or between bacteria and environmental factors. These results indicated that species-species interactions between bacteria play a more important role in regulating community stability than do their interactions with eukaryotic organisms or the environment.

Instead of examining communities taxonomically, an alternative technique is to identify environmentally restricted functional genes and pathways in order to detect the metabolic activities in distinct environments (e.g. Dinsdale et al. 2008; Rusch et al. 2007). Gianoulis et al. (2009) suggested that focusing on the molecular processes of the ecosystem as a whole provided more information than focusing on species composition. Similarly, Barberán et al. (2012) found significant differences between the community profile derived from the 16S rRNA gene and from the functional trait set in 53 metagenomic aquatic samples from the Global Ocean Sampling expedition. The traits these authors analyzed proved to be valuable ecological markers because they discriminated between different marine ecosystems and even between the same ecosystem in different oceans. Moreover, while characterizing the inter-trait relationships, Barberán et al. (2012) proposed some traits that could be further developed as habitat descriptors during sample processing. All these studies highlight that in some ecosystems, exploring the community as a whole may be a more informative approach than a species-focused approach.

These networks are also useful to predict microbial changes of an ecosystem. Yet the accuracy of predictive modeling approaches depends on the characteristics of the environment and microbial community to be analyzed. For example, the dispersal capacity of an oceanic microbial species is believed to be hampered by landmasses and ocean currents (Marshall et al. 1997), so such models would have to include ocean currents as a factor for microbial diversity prediction in the ocean (Follows and Dutkiewicz 2011). In contrast, the dispersal of microbes in soil seems to be dominated by its structural complexity; the heterogeneous structure of soils is believed to impede the dispersal of microbial metabolites (Luttge 2005), so soil microbial community modeling will have to reflect this aspect (Zhang et al. 2005).

Finally, in order to model a dynamic system, other interactions, besides species-species interactions and community-environment interactions, should be taken into account. On the microscale level, each cell also interacts with its cellular environment via protein–protein interaction, protein–DNA interaction, gene interaction, and so on. Furthermore, interaction can be thought of as interchanging genetic material, for example, through viral gene transfer, horizontal gene transfer, or transposon movement. At the macroscale level, communities are not closed and isolated entities; instead, dispersal and interactions between communities and interdependencies of local interactions occur. The “metacommunity” concept, from plant and animal ecology, describes a set of local communities linked by the dispersal of multiple potentially interacting species such that both local interactions and regional processes influence local community assembly (Leibold et al. 2004). While metagenomics has yet to work with such theoretical approaches, metacommunity analysis appears to be an inevitable development of single community studies in the microbial world.

## Conclusions

How has metagenomics influenced our understanding of microbial ecology? Alternatively, what do we know now that we didn’t know when we were just exploring the microbial physiology of cultured bacteria? It is not presumptuous to say that metagenomics has radically altered our understanding of how many bacterial species exist in the environment, and how diverse their genomic potential is. In the last 30 years, through the use of environmental DNA studies and the more recently developed metagenomic techniques, microbial ecologists have changed our perspective regarding microbial biogeography, metabolic interactions, and community dynamics. This was achieved by leveraging technology to reach deep into the microbial world and uncover the dark potential (the previously unknown functionality) of the cells and populations that exist there. The metaphor of illuminating the ‘black box’ of biology is probably highly appropriate to describe the manner in which ‘omics is shining a light on the components of natural communities. Now we have identified some of these components, we can begin characterizing their functional role in the ecosystem.

In many ways metagenomics is at a crossroads between researching the individual components of microbial complexity and the emergent properties of systems. While some commentators have labeled metagenomics as a ‘top-down’ driven methodology that simplifies the complexity of the individual components of the system, it is also a valuable tool for elucidating these components. The gene-centric perspective of metagenomics might seem like a method for exploring the complexity of the system, but the current limitations associated with the annotation of specific potential gene sequences, and the inability to often associate functions with phylogeny, means that metagenomics frequently ends up as a noise-generation machine. Indeed many of the developments in bioinformatics since the inception of NGS have revolved around trying to ‘make sense’ of the hubbub of data. However, metagenomics is now entering a new phase whereby whole pan-genomes are being reassembled from the “noise” in an attempt to reinstate the genome, suitably reconceived, as a unit of information that can contribute effectively to community-level analysis. This research avenue will undoubtedly help us to reconstruct the mechanistic components of metabolic interaction and cell–cell interaction within microbial ecosystems, and may help to predict certain emergent properties of these systems.

All the while as we try to better understand the units of complexity in microbial communities, we are also attempting to use the existing data streams to identify the emergent properties of the community interactions by mapping the dynamic turnover of genes and transcripts, proteins and metabolites on to our existing, if limited, understanding of the metabolic complexity of microbial interactions. This is proving to be valuable for coarse-scale interpretations of the emergent metabolic properties of the highly complex and spatiotemporally dynamic processes that act at the molecular level but are felt by us at the extreme macro-scale. These micro-scale processes influence macro-scale climate processes and ecosystem services that influence human beings and our social cohesion. Therefore, the role of ‘omic approaches (metagenomics, metatranscriptomics and metaproteomics) in elucidating both the micro- and macro-scale dynamics of microbial ecosystems cannot be underestimated, as they have the capability, when combined with appropriate experimental design, to redefine our understanding of how ecosystems respond and feed back to produce system change. These developments, actual and still potential, even with caveats about the challenges ahead, suggest how far beyond the genome community-level analysis has gone and can go.

## Footnotes

1 Western Channel Observatory L4, http://www.westernchannelobservatory.org.uk; the Hawaiian Ocean Time Series, http://hahana.soest.hawaii.edu/hot; the Bermudan Ocean Time Series, http://bats.bios.edu; and National Ecological Observatory Network, http://www.neoninc.org.
